# Gamma Hydroxybutyric Acid (GHB) for the Treatment of Alcohol Dependence: A Review

**DOI:** 10.3390/ijerph6061917

**Published:** 2009-06-24

**Authors:** Fabio Caputo, Teo Vignoli, Icro Maremmani, Mauro Bernardi, Giorgio Zoli

**Affiliations:** 1 Department of Internal Medicine, SS Annunziata Hospital, Cento (Ferrara), Italy; E-Mail: g.zoli@ausl.fe.it; 2 “G. Fontana” Centre for the Study and Multidisciplinary Treatment of Alcohol Addiction, Department of Clinical Medicine, University of Bologna, Italy; E-Mails: tvignoli@yahoo.com (T.V.); mauro.bernardi@unibo.it (M.B.); 3 Vincent P. Dole Dual Diagnosis Unit, Santa Chiara University Hospital, Department of Psychiatry, NPB, University of Pisa, Italy; E-Mail: info@aucns.org

**Keywords:** gamma-hydroxybutyric acid, alcohol withdrawal syndrome, anti-craving drug

## Abstract

Gamma-hydroxybutyric acid (GHB) is a short-chain fatty acid structurally similar to the inhibitory neurotransmitter γ-aminobutyric acid. Clinical trials have demonstrated that 50–100 mg/kg of GHB fractioned into three or six daily doses is able to suppress alcohol withdrawal symptoms and facilitates the maintenance of abstinence from alcohol. These studies have also shown that GHB craving episodes are a very limited phenomenon (about 10–15%). Thus, physicians with access should consider the clinical efficacy of GHB as a valid pharmacological tool for the treatment of alcohol addiction.

## 1. Introduction

Gamma hydroxybutyric acid (GHB) was synthesized in 1960 in an attempt to create an analogue of the ubiquitous inhibitory brain neurotransmitter gamma-aminobutyric acid (GABA) that would cross the blood-brain barrier [1–3]. GHB was first developed as a central nervous system depressant [4,5] and used as an anesthetic adjuvant for minor surgical procedures in laboratory as well as in clinical settings [6–11]. The use of GHB as an anesthetic is now decreasing, even though it is still approved in Germany for intravenous anesthesia [1]. In the 1970s, GHB was found to be effective in the treatment of narcolepsy [12–15]. In particular, nightly doses of GHB were shown to improve the structure of sleep in narcoleptic patients, reducing the number of nocturnal awakenings and daytime attacks of cataplexy [16–18]. In the United States, through a limited distribution program, the FDA approved GHB as a Schedule III Controlled Substance to treat a small subset of patients with narcolepsy who have episodes of weak or paralyzed muscles (i.e. cataplexy) [19]. In addition, since 1992, GHB has been approved in Italy and Austria as a treatment for alcohol dependence [20].

## 2. Metabolism

**Figure 1.**
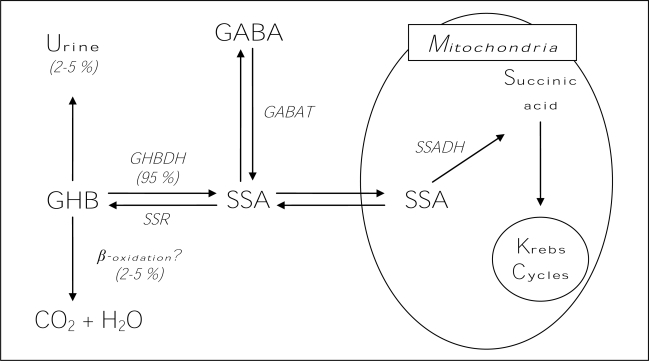
GHB metabolism in the central nervous system. GHBDH: GHB dehydrogenase; SSR: succinic semialdehyde reductase; SSA: succinic semialdehyde; GABA: gamma-aminobutyric acid; GABAT: GABA-transaminase; SSADH: succinic semialdehyde dehydrogenase.

GHB occurs naturally in mammalian brain tissue [21]. The primary precursor of GHB in the brain is GABA, which is transformed into succinic semialdehyde (SSA) through a GABA-transaminase and then converted into GHB via a specific succinic semialdehyde reductase (SSR). GHB can be reconverted to SSA via a GHB dehydrogenase, and then SSA can be converted back to GABA (Figure 1); SSA can also be transformed by succinic semialdehyde dehydrogenase (SSADH) into succinic acid and then further metabolized via the Krebs Cycle in mitochondria (Figure 1). GHB is primarily eliminated by the liver, and only a modest quantity of it remains unmodified (2–5%) and eliminated with urine and/or by a still not fully ascertained process of beta-oxidation (Figure 1) [21].

Exogenous GHB is rapidly absorbed by the gastro-intestinal tract; its peak plasma concentration appears after 15–45 minutes, and its clinical effects after 15–20 minutes. This drug has a dose-dependent elimination half-life; in healthy subjects this can vary from 20 to 53 minutes [22].

## 3. Neuro-Modulatory Properties

In the central nervous system, GHB binds to GHB and GABA_B_ receptors with high and low affinity, respectively [21]. The endogenous neurobiological activity of GHB is mediated through the GHB receptor, while many of the pharmacological and clinical effects of exogenously administered GHB appear to be mediated through the GABA_B_ receptor, where GHB may act both directly, as a partial GABA_B_ receptor agonist, and indirectly through GHB-derived GABA [1,21]. Irrespective of the brain GHB concentration, it is far from certain that GHB interacts directly with the GABA_A_ receptors [1,21,22]. However, the conversion of exogenously administered GHB to GABA induces an activation of GABA_B_ receptor and GABA_A_ receptors too [1,21,22] and this is responsible of GHB sedative and anxiolitic effects.

**Figure 2.**
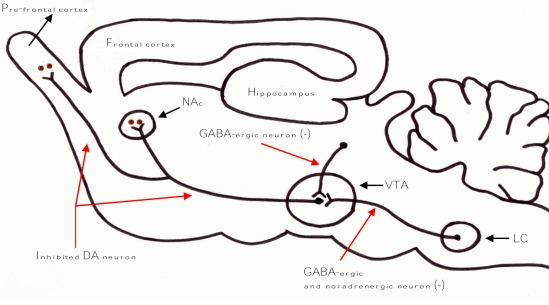

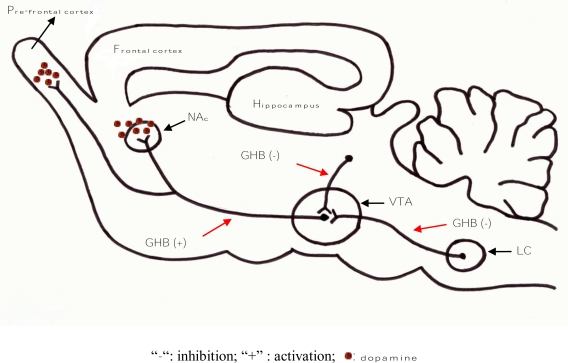
Activity of meso-corticolimbic system in physiological condition (a), and during exogenous administration of GHB (b). (a) dopaminergic neurons (DA) originate from ventral tegmental area (VTA) and project their fibres to the nucleus accumbens (NAc) and to the pre-frontal cortex; DA neurons play a relevant role in physiological reward (i.e. food, sleeping, sexual activity); this circuit is often inhibited by noradrenergic and GABA-ergic neurons originated from the locus ceruleus (LC). (b) GHB induces dis-inhibition of DA originated from VTA through DA direct activation and inhibition of GABA-ergic and noradrenergic neurons with a consequent increase in dopamine release from NAc and pre-frontal cortex; this mechanism is on the basis of the alcohol-mimicking effect of GHB.

Physiologically, the mesocorticolimbic dopaminergic neurons (DA) are involved in reward-dependent learning (Figure 2a). DA have their cell bodies in the ventral tegmental area (VTA) and project into the basal forebrain structures, such as the nucleus accumbens (NAc), amygdala, and frontal and limbic cortexes [21]. Activation of DA, with a resultant increase in the output of dopamine in innervated projection structures, has been reported with virtually all major drugs of abuse. It is supposed that the alcohol-mimetic effect of GHB appears to be related to the effects of the dopamine increase mediated by GABA_B_ receptors in mesocorticolimbic circuitry [21].

Both endogenous and exogenous forms of GHB have a dual action on the GHB receptors and the GABA_B_ receptors. GHB that binds with high affinity to the pre-synaptic GHB receptors decreases the release of GABA, while GHB that binds with a low-affinity site on the GABA_B_ receptors increases activation of cell-surface receptors. Therefore, the administration of exogenous GHB is primarily able to decrease the release of GABA from the pre-synaptic GABA-ergic neurons through effects mediated by a direct activation of GHB receptors [21]. The result would be disinhibition of DA of the VTA with increased dopamine within that circuitry, and this is responsible of the alcohol mimic effect of GHB (Figure 2b) [21]. Finally, GHB has been recently shown to decrease the activity of neurons in the locus ceruleus (LC), providing yet another route by which GHB could disinhibit mesocorticolimbic DA (Figure 2b) [21].

In summary, data indicate that *sedative effect* of exogenously administered GHB (high dose) may be due to a direct effect on GABA_B_ and indirect on GABA_A_ receptors: mainly 100 mg/kg/day to suppress alcohol withdrawal syndrome (AWS) and from 4 to 9 g/day to treat cataplexy in narcoleptic patients. On the other hand, the *alcohol-mimic mechanism* of exogenously administered GHB (low dose) may be due to a decrease in the release of GABA through the effects mediated by GHB receptors on pre-synaptic GABA-ergic and noradrenergic neurons, with a resultant disinhibition of DA and increase in dopaminergic activity in the mesocorticolimbic circuitry (Figure 2b) [21]: mainly 50 mg/kg/day to suppress craving for alcohol intake.

## 4. GHB for the Treatment of Alcohol Withdrawal Syndrome

AWS is mediated by a reduced GABA-ergic activity in the central nervous system [25–27]. Exogenous GHB suppresses AWS symptoms in humans by an indirect activation of GABA_A_ receptors due to the conversion of GHB to GABA [21]. Such a GABA-ergic activity triggers chloride transport across the neuronal membrane, thus inducing a decreased neuronal excitability [28] with consequent resolution of AWS symptoms.

In the clinical area, the efficacy of a non-benzodiazepine GABA-ergic compound such as GHB in suppressing AWS is well demonstrated [29]. After the first pilot study [30], a single-blind trial comparing GHB versus diazepam did not show a significant different efficacy of these drugs in suppressing AWS [31], even though GHB reduced anxiety, agitation and current depression more rapidly than diazepam [31]. A more recent study, however, demonstrated that GHB was even more effective than diazepam in treating AWS [32]. GHB has also been found to be equally efficient as clomethiazole [33], and its efficacy was further confirmed by treating AWS in almost three hundred hospitalized patients affected by different conditions, such as for medical, neurological or psychiatric diseases, trauma or surgery [34]. In all these studies, GHB was employed at the dose of 50–100 mg/kg divided into three or four daily administrations, and no serious side effects were reported.

## 5. GHB as an Anti-Craving Drug in the Maintenance of Alcohol Abstinence

### 5.1. Studies with GHB as Mono-Therapy

As mentioned above, GHB exerts an ethanol-mimicking effect on the central nervous system [35–37]. Consistent with this rationale, GHB has been shown to be capable of inhibiting voluntary ethanol consumption in rats that have a preference for ethanol [35,37,38]. In humans, a randomized double-blind study treating patients with GHB at dose of 50 mg/kg (divided into three daily administrations) or placebo for three months showed that GHB was significantly superior to placebo in increasing the number of abstinent days, in reducing the number of daily drinks and in reducing alcohol craving [39]. Another open multi-centre study confirmed the efficacy of GHB in improving the abstinence rate and in reducing craving for alcohol [40]. GHB also proved to be manageable, with few side-effects, such as dizziness, sleepiness and tiredness early on during treatment (usually resolved after 2–3 weeks). Despite these results, about 30–40% of alcoholics treated with GHB fail to achieve complete abstinence from alcohol even though they sometimes describe a temporary reduction of alcohol craving. Taking into account the short half-life of GHB [22,23], a study was, then, conducted to investigate the efficacy of the administration of a greater fractioning (six times a day) of the same dose (50 mg/kg) of GHB in those subjects who have not achieved alcohol abstinence after the administration of three daily doses of this drug [41]. The results showed a significant reduction of alcohol craving in a greater percentage of alcoholics who were able to achieve complete abstinence from alcohol.

More recently, a one-year open-label study tested the efficacy of GHB (doses ranging between 25 and 100 mg/kg/day) in “treatment-resistant” chronic alcoholics defined as patients who have previously followed at least two attempts at treatment [i.e. use of psychoactive drugs such as selective serotonin reuptake inhibitors, mood stabilizers, tricycles and/or self-help group intervention] without achieving alcohol abstinence or those who relapsed into heavy drinking during attendance at self-help groups or who were not helped in achieving alcohol abstinence by their precarious psycho-social or environmental conditions [42]. The results of the study showed that 60% of patients were “responders”, i.e., patients who successfully achieved complete abstinence from alcohol together with social adjustment (full-responders) or patients who reduced their alcohol intake but did not accomplish complete alcohol abstinence (partial-responders) [42]. The retention rate during treatment with GHB was, therefore, significantly higher than the retention rate of the same sample treated with previous pharmaco-therapies. Furthermore, this study confirmed that the only significant predictor of the retention rate was the six-times/daily fractionated administration of GHB [42], a result in close agreement with the previous study [41].

Laboratory studies with healthy subjects investigating the effects of administering alcohol and a single dose of GHB 50 mg/kg together showed an increased rate of side-effects, probably due to the combination of GHB and alcohol [43]. This effect was not observed in the clinical studies with alcoholics reported above. In fact, no side effects due to the combination of GHB 50 mg/kg (divided into 3 to 6 daily administration) and alcohol were observed in those GHB-treated alcoholics who were still drinking during the treatment [39–41,44,45]. It is conceivable that the use of the same dose of 50 mg/kg divided into 3–6 daily administrations was able to prevent the occurrence of unsafe effects when associated with ethanol [46].

### 5.2. Comparative Studies

A 3-month open randomized comparative study evaluating the efficacy of oral doses of GHB (50 mg/kg of body weight t.i.d) compared with oral doses of naltrexone (NTX) (50 mg/day) in maintaining abstinence from alcohol in patients mostly with moderate dependence showed a better efficacy with GHB than NTX (66.7% vs 35.3%, P < 0.02) [44]. Nevertheless, in the same study, in the NTX group patients who failed to be abstinent did not relapse into heavy drinking, confirming the ability of this drug to reduce alcohol relapses, while in the GHB group all the patients who did not maintain abstinence relapsed into heavy drinking (∼11%). Craving for GHB was not observed, and no sedative additive effects due to alcohol and GHB interaction in patients who relapsed, drug withdrawal syndrome or side effects due to drug suspension on drug discontinuation were shown. Moreover, a 12-month comparative study showed that, despite a trend in favor of GHB, this drug administered at a dose of 50 mg/kg/day, NTX at a dose of 50 mg/day, and disulfiram at a dose of 200 mg/day proved to have a similar effect in maintaining alcohol abstinence at the end of treatment, 65%, 49% and 40%, respectively [47]. None of the patients belonging to the GHB group developed craving for this drug, and side-effects were well tolerated in all groups.

### 5.3. Combined Studies

Considering the data emerging from the comparative studies, one hypothesis arose: are GHB and NTX able to work better if combined together, taking advantage of the former’s alcohol-mimicking effect and the latter’s anti-reward property? In order to confirm this suggestion, a 3-month randomized study was performed in patients mostly with severe alcohol dependence [45]. In this 3-month open randomized comparative study, the combined treatment of GHB and NTX was shown to be more effective in maintaining abstinence from alcohol than GHB and NTX used singly, 72.2%, 40% and 5.9%, respectively [45]. The number of relapses into heavy drinking also tended to occur less frequently in the combination group (no cases) than in either the GHB group or the NTX group. These data support the above-mentioned preliminary hypothesis suggesting that the two drugs may combine their different actions synergistically without suppressing the favorable effects of each other. In addition, as demonstrated by the absence of patients who developed craving for GHB in the combined group with respect to the group taking GHB alone (∼10%) [45], it may be hypothesized that a modulation effect induced by the anti-reward property of NTX is able to avoid the onset of craving for GHB as previously also demonstrated by three clinical experiences [48]. Another 6-month open randomized study evaluating whether GHB or NTX or its combination could help to maintain alcohol abstinence in patients following a treatment with escitalopram, an anti-depressant agent belonging to the category of selective serotonin reuptake inhibitors, has also been performed [49]. In this study the combination therapy in association with escitalopram proved to be more efficient in preventing alcohol relapses than GHB plus escitalopram, NTX plus escitalopram or escitalopram given alone, 83.3%, 50%, 33.3%, and 18.1%, respectively [49]. In fact, the craving mechanism implicated in alcohol addiction may differ in different subtypes of alcohol-dependent patients [50,51]. In particular, dopaminergic/opioidergic deregulation has been implicated in reward craving, GABA ergic/glutamatergic deregulation in relief craving, and serotonergic deregulation in obsessive craving [50–52]. It is possible that different craving conditions and profiles co-exist in a single patient, which is why drug combinations (association of GHB, NTX and escitalopram) could be more effective than mono-therapy in reducing relapses. An understanding of the different forms of craving could have important implications particularly in the field of anti-craving drug therapy.

## 6. Craving for and Abuse of GHB in Clinical Studies

Craving for and abuse of GHB remains one of the crucial points during the use of this drug. However, episodes of craving for GHB in alcoholics, when manifested, are a very limited phenomenon (about 10%) [53]; on the other hand, it is to be hoped that better manageability and safety of GHB can probably be improved with the identification of groups of alcoholics more predisposed to develop this unfavorable effect. A study investigating the risk of developing craving for and abuse of GHB among different types of alcoholics was, therefore, performed [54]. In this study, indeed, 47 patients were enrolled and divided into four different sub-types of alcoholics, and treated with an oral dose of GHB (50 mg/kg of body weight fractioned into three daily administrations) for three months. At the end of the study, besides a general efficacy of GHB in maintaining alcohol abstinence in all four groups of patients, craving for GHB was statistically significantly higher in those alcoholics with previous cocaine dependence than in “pure” alcoholics (patients with a diagnosis of alcohol dependence without other addictive disorders) (90% vs 14.3%, P < 0.001), with 60% abuse of it [54]; however, craving for GHB did not differ between “pure” alcoholics and alcoholics with previous heroin dependence except for the fact that all alcoholics with previous heroin dependence abused GHB, while none of the “pure” alcoholics manifested this addiction. The greater incidence of craving for and abuse of GHB in alcoholics with a previous diagnosis of cocaine or heroin dependence is an interesting finding and may be putatively explained by the alteration of dopamine system [55]. It has been clearly described that patients with previous chronic exposure to heroin or cocaine may present a down-regulation of D_1_ and D_2_ receptors, and only high doses of GHB (> 50 mg/kg/die) may exert a reward effect [56,57]. Moreover, imaging studies with positron emission tomography have shown that chronic exposure to cocaine, alcohol or opiates down regulates D_2_ receptors in striatum, and these adaptations may persist for a long time after substance cessation [58]. In addition, chronic exposure to cocaine reduces GABA_B_ receptor activity in the meso-cortico-limbic area [59,60]. The consequence is a reduction in dopamine release, which persists after cocaine cessation. As GHB acts on GABA_B_ receptors, it is likely that alcoholics with previous cocaine addiction present a down-regulated GABA_B_ system and are more predisposed to develop craving and episodes of abuse of GHB. Thus, GHB, a GABA_B_ receptor agonist, may act as a substitutive drug, so that alcoholics with previous cocaine or heroin addiction tend to become predisposed to misuse of the drug. Interestingly, in the above mentioned study [54], alcoholics following a methadone maintenance treatment (MMT) program did not develop craving for GHB. As some recent studies have shown that MMT may induce a μ-opioid receptor desensitization in rats [61] and a long-lasting striatum dopamine neuron impairment in heroin users [62], a dopamine independent pathway may be responsible for these results; the low percentage of subjects who failed to maintain abstinence for alcohol in the MMT group may confirm this hypothesis.

## 7. Conclusions

Available studies have demonstrated that GHB appears to be effective both in the management of AWS and in the maintenance of long-term abstinence from alcohol [63,64]. Moreover, it is worth noting that all the cited clinical trials have clearly demonstrated that GHB discontinuation is not followed by withdrawal syndrome, irrespective of its use for treating AWS or maintaining abstinence from alcohol; therefore, the discontinuation of GHB does not require a tapering procedure [65]. None of the above-mentioned trials reported serious side effects during the treatment of GHB; in addition, the fractioning of the GHB doses, from three to six daily administrations, avoided the occurrence of additive sedative effects in patients who voluntarily use alcohol during the treatment with this drug [44–46]. Moreover, due to its short half-life (4–6 hours) [23], GHB may be also safe in patients with decompensated liver disease with ascites effusion [24]; however, this data needs to be confirmed by further clinical trials. Furthermore, even though rare and transitory episodes of sedation due to GHB abuse have been reported, no cases of intoxication, coma or deaths have occurred when the drug is administered under a medical supervision [40,54]. On the other hand, when GHB is used as a recreational drug of abuse (non-clinical use), several cases of intoxication and even death after a single and self-administered dose of the “street” formulation have been reported [66–68].

In conclusion, we believe that specialists in the treatment of alcohol addiction should be less concerned by the risk of GHB intoxication when this drug is employed for treating alcoholism under close medical surveillance. They should not be discouraged from using GHB in the treatment of alcohol addiction, as long as some rules are followed during its administration: a) not exceeding 50–100 mg/kg fractioned into three to six daily administrations; b) using GHB only for the treatment of pure alcoholics, and avoiding this drug for those alcoholics with previous cocaine or heroin dependence; c) planning strict medical surveillance (weekly visits) and designating a family member to whom GHB should be entrusted [69]. The safety and efficacy of GHB, thus, need to be emphasized, as this drug represents a most useful tool for treating alcohol dependence [70,71].
